# Supplementary material for: Identification of photoperiod-induced specific miRNAs in the adrenal glands of Sunite sheep (Ovis aries)
Source: Front Vet Sci. 2022 Jul 22;9:888207. doi: 10.3389/fvets.2022.888207 (PMC9354845; doi:10.3389/fvets.2022.888207)
Supplement: Supplementary file 1 [file Data_Sheet_1.ZIP › Supplementary file/miRDeep2 file.zip!/miRDeep2 file/Know_Structure/SPLP42A3/oar-mir-433.pdf]

miRBase precursor : oar-mir-433
Total read count : 1046
oar-miR-433-5p read count : 1
oar-miR-433-3p read count : 1045
remaining reads : 0

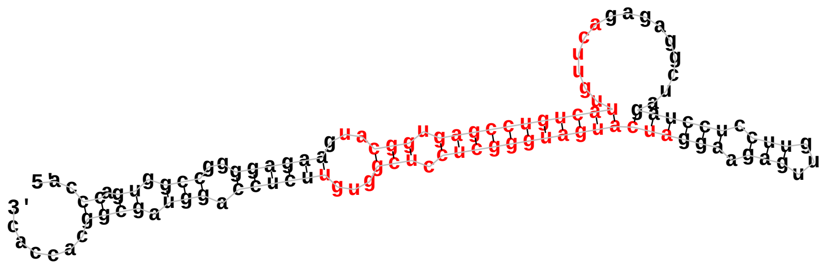

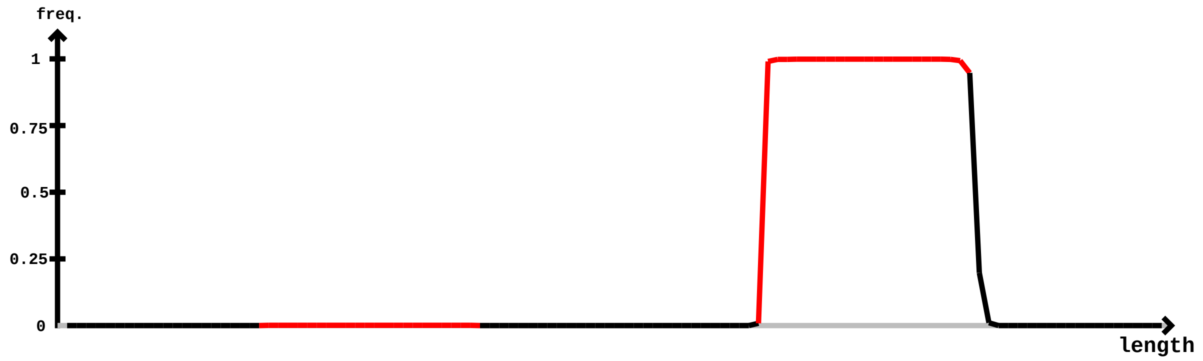

oar-miR-433-5p

oar-miR-433-3p

5'- acccaguggccggggagaaguacggugagccugucauuguucagagaggcuagauccuccuuguugagaaggaucaugaugggcuccucgguguucuccagguagcggcaccac -3'	exp		
..((.((.(((..((((((...(((.(((((((((((...............((((((.(((...))).))))))))))))))))))))).)))...)))))).))).))))..........	reads	mm	sample
....................uacggugagccugucauuguuc..	1	0	seq
....gaucaugaugggcuccucggug.....................	1	0	seq
....gaucaugaugggcuccucggugu....................	8	0	seq
....aucaugaugggcuccucgg.......................	1	0	seq
....aucaugaugggcuccucggu......................	5	0	seq
....aucaugaugggcuccucggug.....................	47	0	seq
....aucaugaugggcuccucggugu....................	767	0	seq
....aucaugaugggcuccucgguguu...................	196	0	seq
....aucaugaugggcuccucgguguuc..................	11	0	seq
....ucaugaugggcuccucggugu....................	8	0	seq
....augaugggcuccucggugu....................	1	0	seq
